# Comparison of Mesenchymal Stromal Cells From Different Origins for the Treatment of Graft-vs.-Host-Disease in a Humanized Mouse Model

**DOI:** 10.3389/fimmu.2019.00619

**Published:** 2019-04-02

**Authors:** Céline Grégoire, Caroline Ritacco, Muriel Hannon, Laurence Seidel, Loïc Delens, Ludovic Belle, Sophie Dubois, Sophie Vériter, Chantal Lechanteur, Alexandra Briquet, Sophie Servais, Gregory Ehx, Yves Beguin, Frédéric Baron

**Affiliations:** ^1^Hematology Research Unit, GIGA-I3, GIGA Institute, University of Liège, Liège, Belgium; ^2^Department of Clinical Hematology, University Hospital Center of Liège, Liège, Belgium; ^3^Department of Biostatistics, SIMÉ, University Hospital Center of Liège, Liège, Belgium; ^4^Endocrine Cell Therapy, Centre of Tissue and Cellular Therapy, Cliniques Universitaires Saint-Luc, Brussels, Belgium; ^5^Laboratory of Cell and Gene Therapy, University Hospital Center and University of Liège, Liège, Belgium

**Keywords:** mesenchymal stromal cells, graft-vs.-host-disease, hematopoietic stem cell transplantation, xenogeneic, NSG, bone marrow, umbilical cord, adipose tissue

## Abstract

Mesenchymal stromal cells (MSCs) have potent immunomodulatory properties that make them an attractive tool against graft- vs.-host disease (GVHD). However, despite promising results in phase I/II studies, bone marrow (BM-) derived MSCs failed to demonstrate their superiority over placebo in the sole phase III trial reported thus far. MSCs from different tissue origins display different characteristics, but their therapeutic benefits have never been directly compared in GVHD. Here, we compared the impact of BM-, umbilical cord (UC-), and adipose-tissue (AT-) derived MSCs on T-cell function *in vitro* and assessed their efficacy for the treatment of GVHD induced by injection of human peripheral blood mononuclear cells in NOD-scid IL-2Rγ^null^ HLA-A2/HHD mice. *In vitro*, resting BM- and AT-MSCs were more potent than UC-MSCs to inhibit lymphocyte proliferation, whereas UC- and AT-MSCs induced a higher regulatory T-cell (CD4^+^CD25^+^FoxP3^+^)/T helper 17 ratio. Interestingly, AT-MSCs and UC-MSCs activated the coagulation pathway at a higher level than BM-MSCs. *In vivo*, AT-MSC infusions were complicated by sudden death in 4 of 16 animals, precluding an analysis of their efficacy. Intravenous MSC infusions (UC- or BM- combined) failed to significantly increase overall survival (OS) in an analysis combining data from 80 mice (hazard ratio [HR] = 0.59, 95% confidence interval [CI] 0.32–1.08, *P* = 0.087). In a sensitivity analysis we also compared OS in control vs. each MSC group separately. The results for the BM-MSC vs. control comparison was HR = 0.63 (95% CI 0.30–1.34, *P* = 0.24) while the figures for the UC-MSC vs. control comparison was HR = 0.56 (95% CI 0.28–1.10, *P* = 0.09). Altogether, these results suggest that MSCs from various origins have different effects on immune cells *in vitro* and *in vivo*. However, none significantly prevented death from GVHD. Finally, our data suggest that the safety profile of AT-MSC and UC-MSC need to be closely monitored given their pro-coagulant activities *in vitro*.

## Introduction

Allogeneic hematopoietic cell transplantation (allo-HCT) has remained the best therapeutic option for many patients with hematological or immune disorders (1). Its efficacy in hematological malignancies depends not only on the chemo/radiotherapy given in the conditioning regimen, but also on graft-vs.-tumor (GvT) effects mediated mainly through donor T cells contained in the graft (2, 3). However, donor T cells can also recognize the tissues of the recipient as foreign, causing graft-vs.-host disease (GVHD) (4–6), a life-threatening complication of allo-HCT (7, 8). The complex physiopathology of acute GVHD involves both innate and adaptive immune activation in response to inflammatory triggers such as damage-associated molecular pattern (DAMP) molecules released from damaged cells or extracellular matrix, and pathogen-associated molecular pattern (PAMP) molecules from bacteria, viruses and fungi. The main effectors of acute GVHD are donor CD4^+^ and CD8^+^ T cells, the latest causing tissue damages through expression of FAS ligand and release of granzyme B, perforin and cytokines such as tumor necrosis factor-alpha (TNFα). CD4^+^ T-cell activation, differentiation, and survival require three signals: (1) interaction of T-cell receptor (TCR) with antigen presenting cells (APCs) expressing host major histocompatibility complex (MHC) and/or host minor histocompatibility antigens, (2) positive costimulatory signals (including CD28, ICOS, CD40L, OX40, 4-1BB) and (3) cytokines such as interleukin (IL)-2, IL-7, IL-15, and polarizing T helper 1 (Th1), Th2 and Th17 cytokines (9). Regulatory mechanisms include mainly regulatory T cells (Tregs; CD4^+^CD25^+^FoxP3^+^), but also type 1 regulatory T cells (Tr1) secreting IL-10, myeloid-derived suppressor cells and tolerogenic dendritic cells (9).

GVHD has remained a serious limitation of allo-HCT (7, 8). Only half of the patients respond to first-line steroid therapy, and the outcome of patients with steroid-refractory GVHD has remained dismal (10). Therefore, there is a real need for new effective strategies to treat acute GVHD.

Mesenchymal stromal cells (MSCs) are multipotent progenitors within the bone marrow capable of differentiating into various cells and tissues, such as chondrocytes, osteoblasts and adipocytes (11). In addition to their support to hematopoiesis, MSCs have demonstrated potent tissue repair abilities and immunomodulatory properties (12, 13). Specifically, MSCs interact with lymphocytes, natural killer (NK) cells and APCs, through release of soluble factors [such as prostaglandin-E2, transforming growth factor beta-1, or human leukocyte antigen [HLA]-G but also, as recently reported, programmed death-ligand [PD-L] 1 and PD-L2 (14)], induction of indoleamine 2,3 dioxygenase (IDO), and/or cell contact signaling (12, 13). Importantly, MSCs have similar immunosuppressive potency against autologous and allogeneic lymphocytes. All these characteristics make them a promising tool against GVHD (15–17).

In the last 2 decades, MSC infusions have been evaluated for both prevention and treatment of GVHD. A number of phase II trials reported lower incidences of acute GVHD in patients co-transplanted with MSCs than in historical or concurrent controls (18–20). However, a meta-analysis of trials of MSC infusion in the setting of GVHD prophylaxis failed to demonstrate a significant impact of MSC infusion on GVHD (21). Among the phase I/II trials assessing the efficacy of allogeneic MSCs for the treatment of steroid refractory acute GVHD, complete response (CR) rates varied between 10 and 75% (22–24), providing a median 6-month survival of 63% (95% CI 50–74%) after MSC infusion in another large meta-analysis (25). Importantly, the sole randomized placebo-controlled phase III trial assessing MSC infusions as treatment for steroid-refractory GVHD reported thus far failed to reach the primary endpoint (increase in the rates of durable [≥28 days] CR) (26). The heterogeneity in the design of these studies as well as the heterogeneity in MSC products used might have participated in the discrepancies between their results. Based on these observations, a recent trial aimed at selecting subjects likely to be responders, in light of the results of the first clinical studies. Early MSC therapy in pediatric gut and/or liver steroid-refractory GVHD seems indeed promising with improvement of overall response at day 28 (CR + partial response [PR]: 69%) (27), although the final results of the trial have not been published yet. MSCs can also display pro-inflammatory properties (including secretion of pro-inflammatory cytokines such as IL-6 and IL-8) that may hamper their efficacy (28). These findings stress the need for more pre-clinical studies aiming to a more thorough understanding of MSC mechanisms of action and parameters of efficacy.

Since their first discovery in bone marrow (BM), MSCs have been successfully isolated from several other tissues, including adipose tissue (AT), umbilical cord (UC), umbilical cord blood, and placenta. MSCs from different sources share many characteristics, but also display many phenotypical and functional differences (29, 30). Although they all exhibit immunomodulatory properties, few studies directly compared their therapeutic benefits. Here, we compared the ability of BM-MSCs, AT-MSCs, and UC-MSCs to treat GVHD in NOD-scid IL-2Rγ^null^ HLA-A2/HHD (NSG-HLA-A2/HHD) mice infused with human peripheral blood mononuclear cells (PBMCs) from non-HLA-A2 donors. We recently demonstrated that GVHD in that humanized model is caused by a limited number of CD4 and CD8 xeno- as well as probably allo- reactive T-cell clones that expand via activation of the TCR, costimulation, IL-2/STAT5, mTOR, and Aurora kinase A pathways and differentiate into effector cells in GVHD-target organs, secreting high amounts of interferon gamma (IFNγ) and TNFα (31). This model mimics some important aspects of GVHD pathogenesis in humans and non-human primates (32).

## Materials and Methods

### Mesenchymal Stromal Cells

BM-MSCs were produced at the Laboratory of Cellular and Genic Therapy (LTCG, CHU Liège, Belgium) under GMP condition as previously described (33). UC-MSCs were isolated in our Hematology Research Unit (GIGA-I3, University of Liège, Belgium). Umbilical cords were provided by the maternity ward of the Center Hospitalier du Bois de l'Abbaye (Liège, Belgium), with informed consent of the mothers. Briefly, umbilical cord segments of approximately 5 cm were cut longitudinally to increase the contact area and plated onto a plastic surface for 5 days in Dulbecco's Modified Eagles Medium–Low Glucose with Glutamax (DMEM-GLX, Fisher-Bioblock, Invitrogen, Merelbeke, Belgium) supplemented with 10% gamma-irradiated Fetal Bovine Serum (FBS, Hyclone, Perbio Sciences, Utah, USA) and antibiotics (Penicillin/Streptomycin [P/S]). After 5 days, the cord segments were removed and the culture was pursued until subconfluency. AT-MSCs were provided by the Endocrine Cell Therapy unit of the Cliniques Universitaires Saint-Luc (Brussels, Belgium), and produced as previously described (34).

All MSCs were cryopreserved at passage 2 or 3, then thawed and cultured 1–2 week(s) before trypsinization and injection to mice or use in *in vitro* experiments.

### MSC / PBSC Co-Cultures

MSCs (1 × 10^4^ or 2 × 10^4^) were plated in flat-bottom 96-well plates (Becton–Dickinson) in RPMI 1,640 medium supplemented with 10% FBS, penicillin (100 U/ml), streptomycin (100 mg/ml), l-glutamine (2 mM) (all from Lonza), sodium pyruvate (100 mM), non-essential amino acids (100 mM), and β-mercaptoethanol (5 × 10^−5^ M) (all from Gibco, Merelbeek, Belgium). For inflammatory stimulation, MSCs were incubated with IFNγ 10 ng/ml and TNFα 15 ng/ml during 40 h before harvest. For PBMC proliferation assays, MSCs were irradiated at 22 Gy using a 137Cs source (GammaCell 40, Nordion, Ontario, Canada) after 4-h incubation to reduce their proliferation. Allogeneic human PBMCs were isolated from blood samples of healthy volunteer donors by Ficoll Paque^R^ Plus density gradient. For lymphocyte proliferation assays, PBMCs were stained with CFSE using a CellTrace CFSE Cell Proliferation Kit (Thermofisher) according to the manufacturer's instructions. PBMCs (1 × 10^5^) were added to wells in a total volume of 200 μl containing or not irradiated MSCs, in the presence of anti-CD3/CD28 microbeads (Invitrogen, Dynal A/S, Oslo, Norway) at a bead/cell ratio of 1:1 in proliferation assays and 1:5 in the other experiments. Recombinant human IL-2 300 U/ml (PeproTech, USA) was added for the regulatory T-cell (Treg) assays. Cells were incubated at 37°C during 3–7 days depending on the assay, and collected at different time points for FACS analysis.

### Humanized Mouse Model of Graft-vs.-Host Disease

All experimental procedures and protocols used in this investigation were reviewed and approved by the Institutional Animal Care and Use Committee of the University of Liège, Belgium (Certification No. 1480). Animal welfare was assessed at least once per day. We used NOD-scid IL-2Rγnull (NSG) mice expressing the HHD construct designed for the expression of human HLA-A0201 covalently bound to human β2 microglobuline (NSG-HLA-A2/HHD) (Jackson laboratory) (35), aged from 8 to 12 weeks at the start of the experiments. Both male and female mice were used, and their repartition was balanced between treatment groups in each cohort. They received a sub-lethal (2 Gy) irradiation (137Cs source gamma-cell irradiator 40, Nordon, Canada) on day−1, followed on day 0 by an intravenous (i.v.) injection (lateral tail vein) of 1 or 1.5 × 10^6^ PBMCs obtained from healthy mismatched (non-HLA-A2) volunteers to induce GVHD. We previously reported that infusion of PBMCs from non-HLA-A2 donors induced stronger GVHD than injection of PBMCs from HLA-A2^+^ donors in NSG-HLA-A2/HHD mice (31). Hence, in this model, GVHD is both xenogeneic (human to mouse) and allogeneic (non-HLA-A2 donor to HLA-A2 recipient). We used PBMCs from 3 different donors for the 3 cohorts to account for inter-donor variability (all groups of mice were transplanted with the same donor within each cohort). Mice (usually 8 per group) were treated with 3 i.v. injections of BM-, UC- or AT- MSCs diluted in 200 μL PBS, or the same volume of PBS (control group) on days 14, 18, and 22. In the second cohort, one group received i.p. injections of 4 mg tocilizumab (RoActemra®, Roche) 2 h before each MSC infusion. GVHD severity was assessed by a scoring system that incorporates four clinical parameters—weight loss, posture (hunching), mobility and anemia—each parameter receiving a score of 0 (absent) to 2 (maximum), as previously described (31, 36, 37). Mice were monitored daily during the experiments and assessed for GVHD score three times a week. Mice reaching a GVHD score of 6/8 were euthanized in agreement with the recommendation of our ethical committee. Final scores for animals reaching the limit score were kept in the data set for the remaining time points (last value carried forward). Blood samples were collected by tail puncture at day 28 and day 42 after human cell transplantation for flow cytometry analysis. If enough blood could be harvested from mice, cells were counted with a Sysmex XS-800i®. In the third cohort, additional blood samples were collected 1 day after the 2nd MSC infusion for cytokine measurements.

### Flow Cytometry

For peripheral blood collected from mice, samples were first depleted of erythrocytes using RBC lysis buffer (eBioscience, San-Diego, CA) according to the manufacturer's instructions. Cells were stained with various combinations of fluorescence-conjugated anti-human antibodies. For surface staining, cells were incubated with surface antibodies for 20 min at 4°C in the dark and washed twice with PBS/3% FBS (Lonza). Intracellular staining was performed by using the FoxP3 Staining Buffer Set (eBioscience), according to the manufacturer's instructions. For intracellular cytokine staining, cells were first stimulated for 4 h at 37°C and 5% CO2 in RPMI supplemented with 10% FBS and in the presence of PMA/ionomycin, brefeldin A and monensin (Cell Stimulation Cocktail + Protein Transport Inhibitors, eBioscience), according to the manufacturer's instructions. Data were acquired on a FACSCanto II or LSRFortessa flow cytometer (Becton Dickinson) and analyzed with the Flowjo software v10.0.7r2 (Tree Star Inc., Ashland, OR). Data from the flow cytometry analyses of blood samples of mice in the third cohort were also analyzed with FlowSOM. Data were compensated, then human CD45^+^ cells were manually gated with FlowJo v10, concatenated within the same group and analyzed with the Bioconductor package FlowSOM.

### Cytokine Measurement

Mouse sera were collected with SST Tubes (BD Microtainer), centrifuged for 10 min, then stored at −80°C. The concentration of human cytokines was determined after 2-fold serum dilution, by using a custom Magnetic Luminex Performance Assay (R&D Systems, USA). Procedures were performed according to the manufacturer's instructions. Results were acquired on Bio-Plex System and analyzed with Bio-Plex Manager Software 4.0 (Biorad Laboratories).

### Rotational Thromboelastometry (ROTEM)

MSCs from 3 different healthy donors (2 donors for AT-MSCs) were thawed and cultured 1 week before the experiment. Samples of blood from 3 healthy volunteers were collected in citrated tubes. PBS was used as a negative control. MSCs were incubated 10 min in citrated whole blood at a concentration of 10^6^ cells/ml, then CaCl_2_ (Star-TEM) was added to the sample and measurements of coagulation activation was made using ROTEM® (NATEM assay) according to the manufacturer's procedure. Samples were kept at 37°C during the procedure.

### Statistical Analyses

Data are presented as individual observations (with or without median) or as median with range. For survival analyses, comparisons between groups were made with the log-rank test and with multivariate Cox models adjusted for experiment (one donor PBMC was used per experiment), mouse gender and mouse weight at transplantation. Survival curves were plotted using Kaplan-Meier estimates. Evolution of GVHD scores over time was analyzed with a repeated ordinal logistic model (GENMOD), with adjustment for experiment, mouse gender, and mouse weight at transplantation. GVHD score at death were carried forward after death. For *in vivo* analyses, comparisons between control group and either BM-MSC, UC-MSCs, or AT-MSC groups were made with one-way analysis of variance tests with Dunnett's *post-hoc* procedure. Analyses were adjusted for experiment. For *in vitro* analyses, comparisons between control group and either BM-, UC-, or AT-MSC groups were made using repeated measure one-way analysis of variance test with Dunnett's *post-hoc* procedure (except for inhibition of lymphocyte proliferation: comparisons between BM-, UC-, and AT-MSC groups were made using repeated measure one-way analysis of variance test with Bonferroni *post-hoc* procedure) and comparisons between resting and primed MSC groups were made with paired *t*-tests. To normalize their distribution, some variables underwent prior logarithmic transformation. Results were considered significant at the 5% level (*p* < 0.05). Statistical analyses were carried out with RStudio v1.1.453 and Graphpad Prism 5.0 (Graphpad Software, USA).

## Results

### Impact of MSCs on PBMC Proliferation *in vitro*

We compared the ability of MSCs to suppress PBMC proliferation *in vitro* at two different MSC/PBMC ratios (1/5 and 1/10). Lymphocytes were stimulated with anti-CD3/CD28 beads, mimicking stimulation by APCs as well as early events occurring in human PBMCs infused in NSG-HLA-A2/HHD mice (31). We repeated the experiment with MSCs from 2 to 3 different donors and PBMCs from 2 to 4 different donors for each MSC donor. Some of these experiments were realized in triplicate, and mean values were used for statistical analysis. BM-, UC-, and AT-MSCs were either resting or primed (BM^*^, UC^*^, and AT^*^) by IFNγ and TNFα. This is relevant since previous reports have demonstrated that these cytokines have a profound impact on MSCs (38–42) and since high levels of IFNγ and TNFα are present in the sera of NSG-HLA-A2/HHD mice infused with human PBMCs (31). The impact of MSCs on PBMC proliferation was calculated as percentage suppression compared with the proliferative response in the positive control without MSCs.

**Figure 1:**
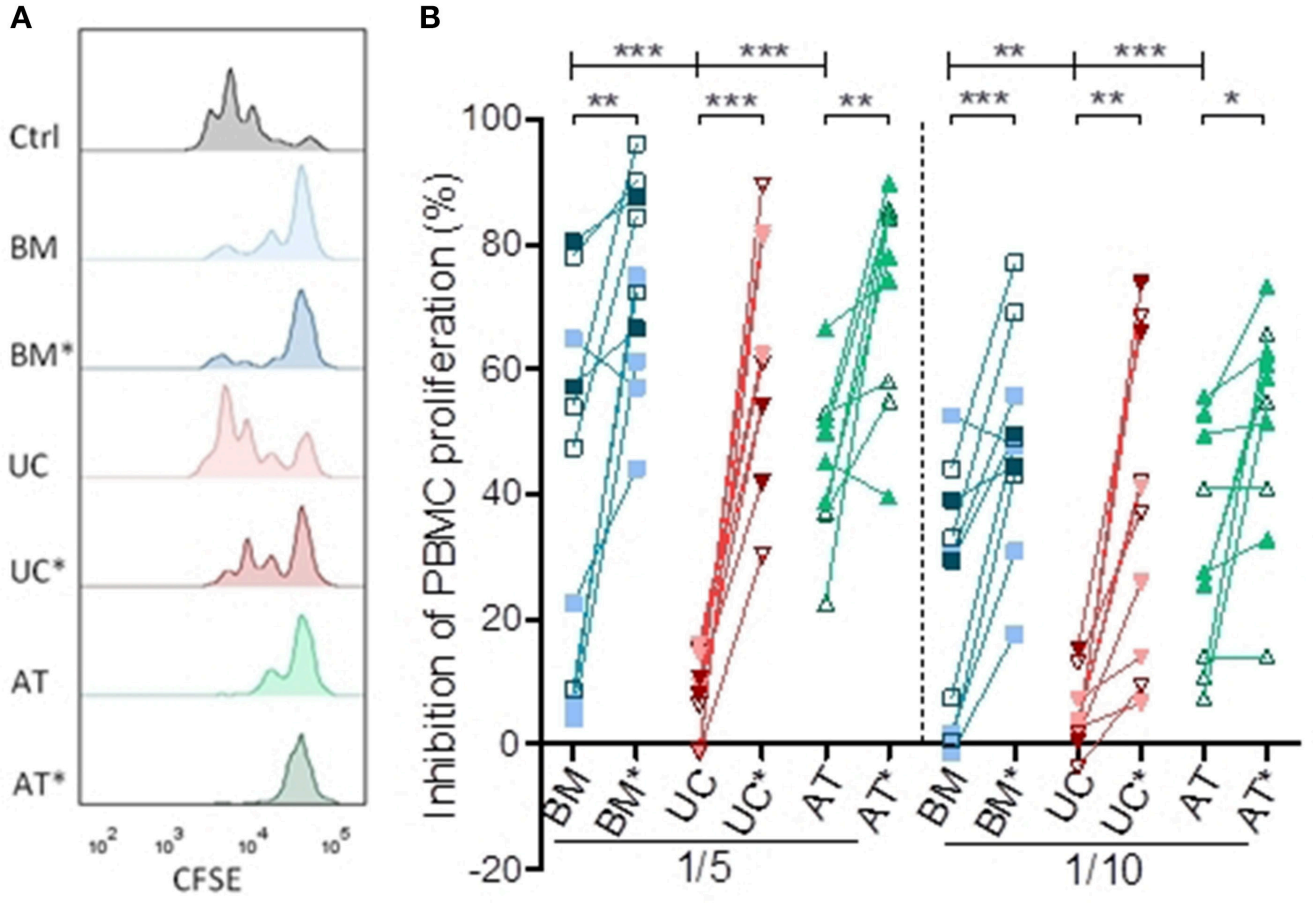
Inhibition of lymphocyte proliferation *in vitro*. PBMCs were cultured with or without MSCs in the presence of anti-CD3/CD28 microbeads for 3 days, at MSC/PMBC ratios of 1/5 and 1/10. Proliferation of PBMCs was assessed using a CellTrace CFSE Cell Proliferation Kit. The effect of MSCs on PBMC stimulation responses was calculated as percentage suppression compared with the proliferative response in the positive control without MSCs. For inflammatory stimulation, MSCs were incubated with IFNγ 10 ng/ml and TNFα 15 ng/ml during 40 h, prior to harvest (BM^*^, AT^*^, and UC^*^). **(A)** Representative plots of PBMC proliferation in coculture with MSCs, assessed by CFSE dilution. **(B)** Inhibition of lymphocyte proliferation. Data are presented as individual observations (or mean value if the experiment was realized in triplicates). White, light, and dark symbols represents MSCs from different donors; each point represents a different MSC-PBMC couple. Differences between resting MSC groups and between primed MSC groups are calculated with repeated measure ANOVA with Bonferroni post-hoc procedures (only results with Bonferroni *post-hoc* tests are represented). Differences between resting and primed MSC groups were calculated with paired *t*-test (^*^p < 0.05, ^**^p < 0.01; ^***^p < 0.001).

After 72 h of co-culture, resting BM- and AT- MSCs were more potent to inhibit PBMC proliferation compared to resting UC-MSCs at a ratio of 1/5 (median inhibition 51 vs. 48 vs. 9%, *p* = 0.0001) and 1/10 (median inhibition 30 vs. 27 vs. 3%, *p* = 0.0005) (Figure 1). As previously observed, BM-, AT- and UC-MSCs primed with IFNγ and TNFα were more potent to inhibit PBMC proliferation than resting MSCs at both MSC/PBMC ratios. Interestingly, primed MSCs from various origins had a comparable potency to inhibit PBMC proliferation (Figure 1). These data suggest that, in the context of CD3/CD28 stimulation, PBMC proliferation is potently inhibited by BM-, UC-, and AT-MSCs primed with IFNγ and TNFα. Without that inflammatory priming, only BM- and AT-MSCs inhibited PBMC proliferation at these low MSC/PBMC ratios.

### Impact of MSCs on Lymphocyte Activation *in vitro*

We also analyzed the effects of BM-, UC-, and AT-MSCs on lymphocyte activation *in vitro*. PBMCs were cultured with MSCs, either resting (BM, UC, and AT-conditions) or primed with IFNγ and TNFα (BM^*^, UC^*^, and AT^*^ conditions), or without MSCs (control condition), at a MSC/PBMC ratio of 1/10. Expression of early (CD69), late (CD25) and very late (HLA-DR) markers of activation of CD4^+^ and CD8^+^ cells was analyzed after 6, 24, 48, 72, and 96 h. The experiment was repeated twice with MSCs and PBMCs from 2 different donors. The kinetics of PBMC activation by anti-CD3/CD28 beads resulted, as previously described (43), in a rapid and brief upregulation of the early activation marker CD69 within 24 h, followed by a rapidly progressive and lasting expression of CD25, and a slowly progressive upregulation of HLA-DR (Supplementary Figure 1).

**Figure 2:**
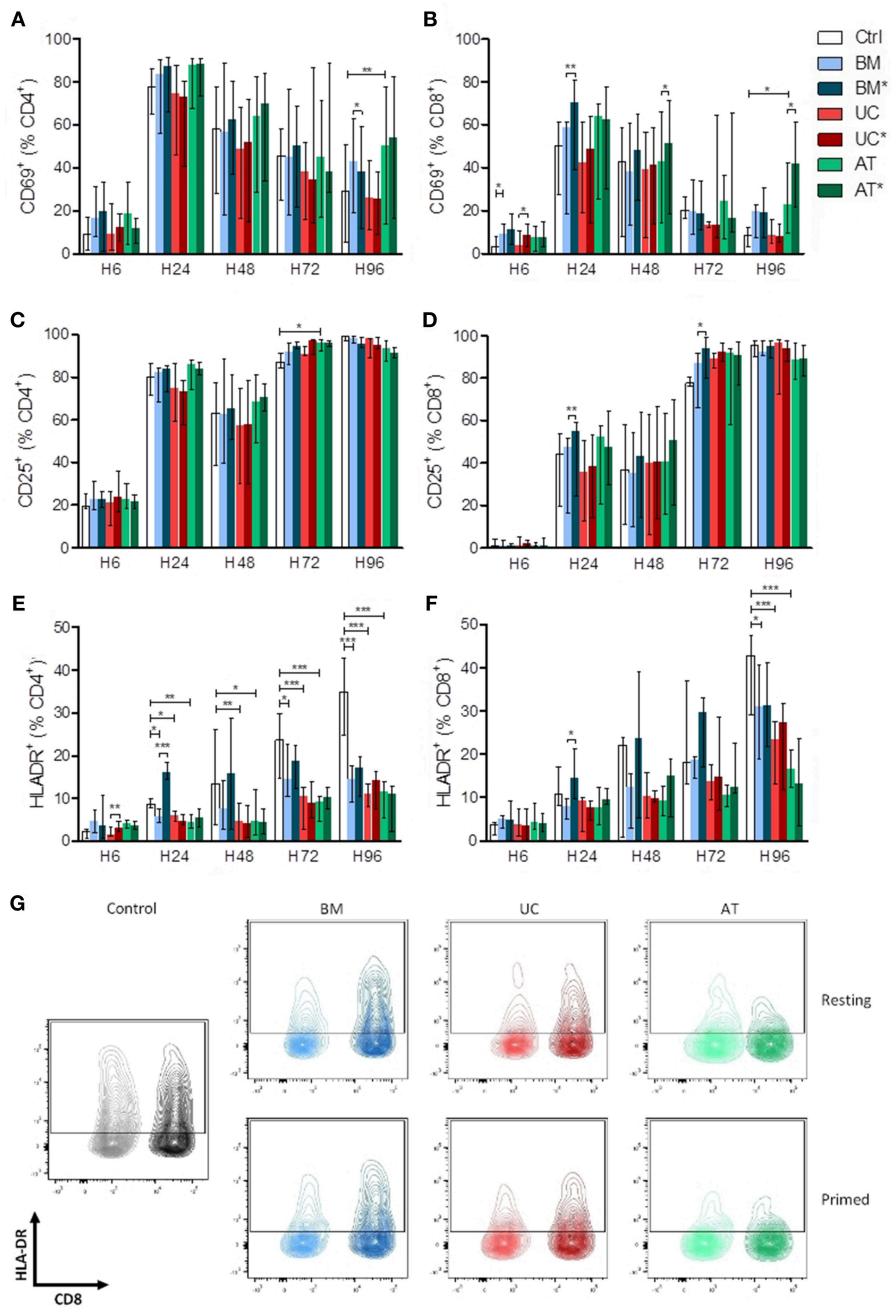
Lymphocyte activation (measured by CD69, CD25, and HLA-DR expression) in co-culture with MSC. PBMCs were cultured with or without MSCs in the presence of anti-CD3/CD28 microbeads for 4 days, at a MSC/PBMC ratio of 1/10. For inflammatory stimulation, MSCs were incubated with IFNγ 10 ng/ml and TNFα 15 ng/ml during 40 h, prior to harvest (BM^*^, AT^*^, and UC^*^). Expression of **(A,B)** CD69, **(C,D)** CD25, and **(E,F)** HLA-DR on CD4^+^ and CD8^+^ lymphocytes was analyzed after 6, 24, 48, 72, and 96 h by FACS. **(G)** Representative plots of HLA-DR expression at H96 in CD4^+^ and CD8^+^ lymphocytes. Data are presented as median with range. Differences between control, and BM, AT, or UC groups are calculated with repeated measure ANOVA with Dunnett's *post-hoc* procedures (only results of Dunnett's *post-hoc* tests are represented). Differences between resting and primed MSC groups were calculated with paired *t*-test (^*^p < 0.05, ^**^p < 0.01; ^***^p < 0.001).

There was no major impact of MSCs on the kinetics of CD69 and CD25 expression on T cells except for a higher expression of CD69 in AT-MSC conditions at 96 h (Figures 2A–D). In contrast, HLA-DR up-regulation on both CD4^+^ and CD8^+^ lymphocytes was clearly impacted by MSC co-culture. Specifically, compared to the control condition, HLA-DR expression on CD4^+^ cells was significantly lower in the BM condition at 24, 72, and 96 h and in UC and AT conditions at 24, 48, 72, and 96 h. The effect of MSC coculture on CD8^+^ cells was less pronounced but was still observed at the latest assessed time point (96 h) (Figures 2E–G). Interestingly, priming of BM-MSCs with IFNγ and TNFα resulted in an early upregulation of HLA-DR on both CD4^+^ and CD8^+^ T cells, while this effect was not observed with primed UC- or AT-MSCs (Figures 2E,F).

Taken together, these data suggest that UC- and AT-MSCs exert a potent inhibitory effect on lymphocyte activation regardless of inflammatory priming, while BM-MSCs elicit transient lymphocyte activation when primed by inflammatory cytokines.

### Impact of MSCs on T Helper Subsets *in vitro*

We also studied the impact of MSC co-culture on lymphocyte subset proportions *in vitro*. We analyzed the effect of BM-, UC-, and AT-MSCs on T helper subset proportions when PBMCs were cultured with anti-CD3/CD28 beads, at a MSC/PBMC ratio of 1/10, for 7 days. For Treg (CD4^+^CD25^+^FoxP3^+^) subset analyses, we added IL-2 in the culture media. The experiment was repeated three times with MSCs and PBMCs from 2 to 4 different donors, and we analyzed the expression of CD25^+^ and FoxP3^+^ as well as IL-10, IFNγ, IL-4, and IL-17 at day 7 (Supplementary Figure 2).

**Figure 3:**
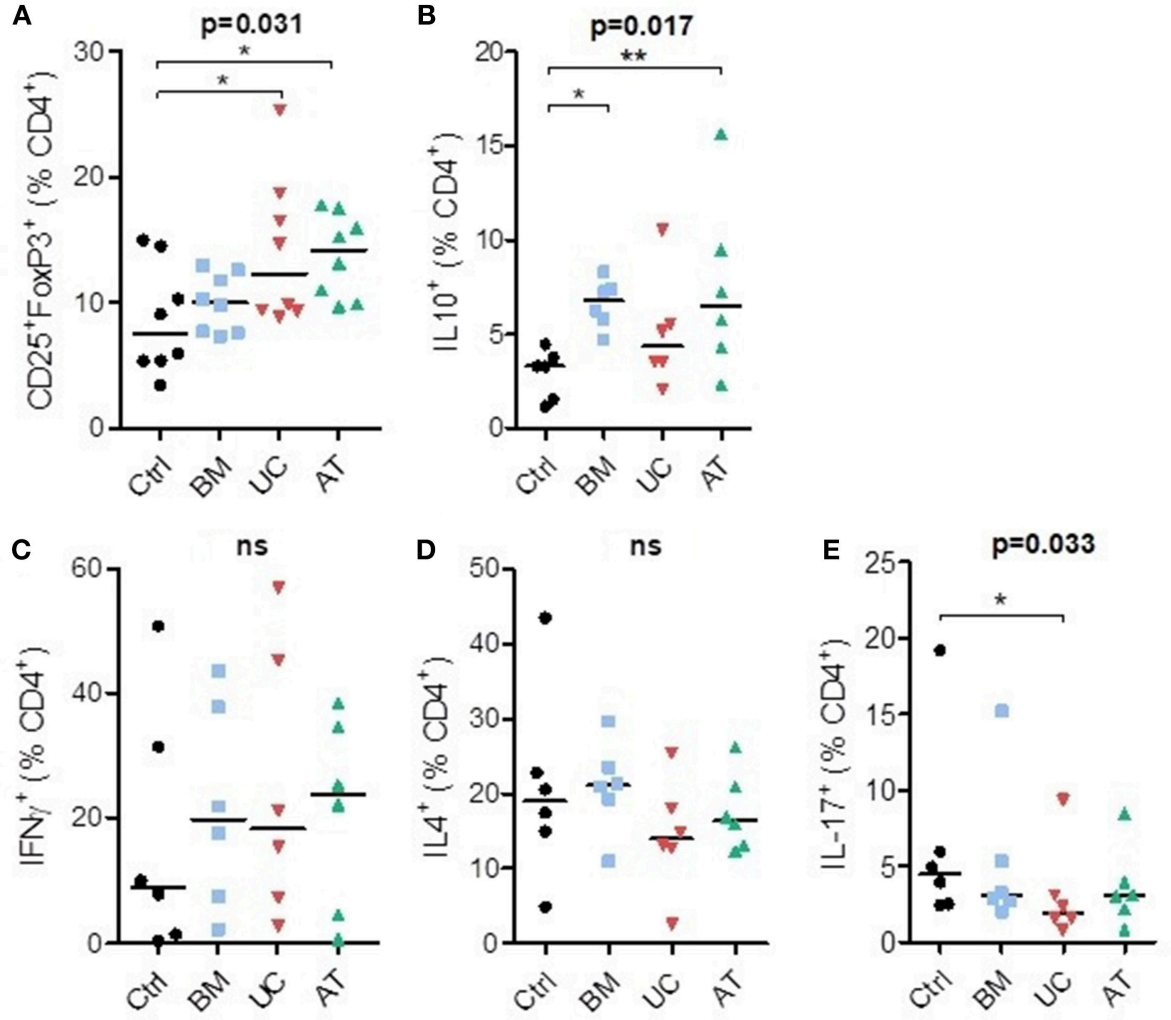
T-helper lymphocyte subsets in co-culture with MSC. PBMCs were cultured with or without MSCs in the presence of anti-CD3/CD28 microbeads (and IL-2 for Treg analyses) for 7 days, at a MSC/PBMC ratio of 1/10. Proportions of **(A)** Treg (CD4^+^CD25^+^FoxP3^+^), **(B)** IL10^+^, **(C)** Th1 (IFNγ^+^), **(D)** Th2 (IL-4^+^), and **(E)** Th17 (IL-17^+^) cells were evaluated at day 7 by FACS. Data are presented as individual observations (or mean value if the experiment was realized in duplicates) with median. Global *p*-values (repeated measure ANOVA-1) are shown as well as comparisons between MSC groups and controls with Dunnett's *post-hoc* procedure (^*^p < 0.05; ^**^p < 0.01).

Co-culture of PBMCs with MSCs increased the percentage of CD4^+^CD25^+^FoxP3^+^ cells (Tregs) at day 7 compared to controls. This reached statistical significance with UC- and AT-MSCs (Figure 3A). Coculture of PBMCs with BM- and AT-MSCs increased the proportion of IL-10^+^ CD4^+^ cells (respectively median 6.8 and 6.5 vs. 3.3% in control condition) (Figure 3B). The proportions of Th1 (IFNγ^+^CD4^+^) and Th2 (IL4^+^CD4^+^) cells were not significantly impacted by MSC coculture compared to the control condition (Figures 3C,D). However, the percentage of Th17 (IL17^+^CD4^+^) cells was lower when PBMCs were cultured with UC-MSCs compared to controls (Figure 3E).

In summary, co-culture of PBMCs with BM-MSCs increased the proportion of IL10^+^CD4^+^ cells, while UC-MSCs resulted in a higher Treg proportion and lower Th17 proportions, and AT-MSCs increased both Tregs and IL10^+^CD4^+^ cells proportions.

### Impact of MSC Therapy on GVHD in Humanized Mouse Model

Mice received sub-lethal (2 Gy) irradiation on day−1, followed by an i.v. injection of PBMCs obtained from healthy mismatched volunteers (non-HLA-A2) on day 0, and 3 i.v. injections of BM-MSCs (BM group), UC-MSCs (UC group), or AT-MSCs (AT group) in 200 μL PBS, or the same volume of PBS alone (control group) on days 14, 18, and 22 (8 mice per group per experiment). In order to prevent inter-donor variability, the experiment was replicated three times with three different donors.

**Figure 4:**
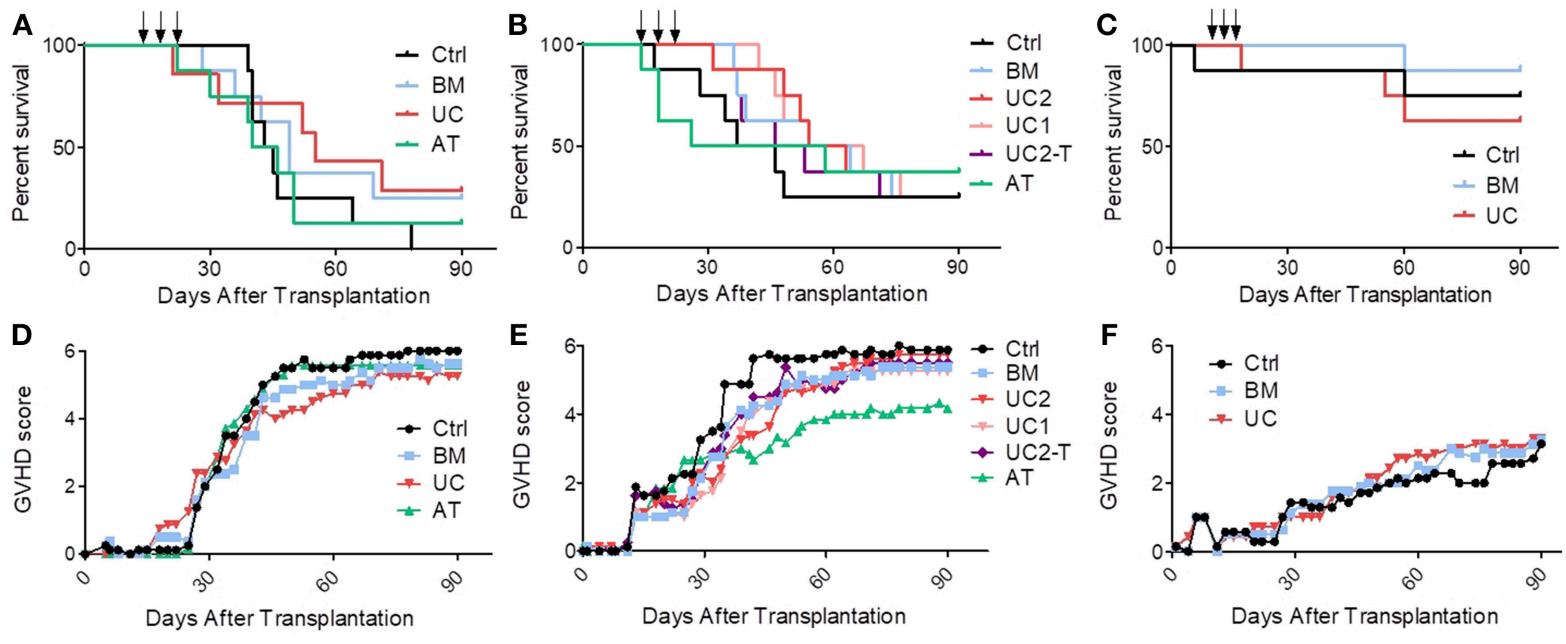
Impact of MSC therapy on GVHD. After 2 Gy total body irradiation, NSG-HLA-A2 mice were transplanted on day 0 with 1–1.5 × 10^6^ PBMCs and treated with 3 i.v., injections (arrows) of 1–2 × 10^6^ MSCs derived from either BM, UC, or AT, or with PBS (control group) on days 14, 18, and 22. **(A)** Survival curves of mice from the 1st cohort (1 × 10^6^ PBMCs − 1 × 10^6^ MSCs); *n* = 8 mice per group. **(B)** Survival curves of mice from the 2nd cohort (1.5 × 10^6^ PBMCs − 2 × 10^6^ MSCs in BM, AT, and UC2 groups, 1 × 10^6^ MSCs in UC1 group — IP infusions of tocilizumab in UC2-T group); *n* = 8 mice per group. **(C)** Survival curves of mice from the 3rd cohort (1 × 10^6^ PBMCs − 1 × 10^6^ MSCs); *n* = 8 mice per group. **(D–F)** GVHD scores of mice from cohorts 1, 2, and 3 (data shown as means).

In the first cohort, mice received 1 × 10^6^ PBMCs, resulting in the development of an acute GVHD that was lethal in all control mice. We started to infuse MSCs (1 × 10^6^ MSCs/dose/mouse) or PBS at day 14, when mice showed the first signs of GVHD. Control mice started to die 15 days after the 3rd infusion (from day 39). We observed an earlier mortality in the MSC groups, especially in the AT group in which one mice died at the time of the third MSC infusion, probably of pulmonary embolism (although no necropsy was performed to prove it). However, UC-MSC therapy eventually resulted in a trend for a longer median survival (63 vs. 44, 49, and 43 days in the control, BM and AT groups, respectively, ns) (Figure 4A).

Given the results of the first cohort, we elected to slightly increase the number of PBMCs infused in order to induce a stronger GVHD. Further, we elected to increase the MSC dose to 2 × 10^6^ MSC/dose/mouse, except for the UC groups in which we compared 1 and 2 × 10^6^ MSC/dose/mouse (UC1 and UC2 groups). Mice received 1.5 × 10^6^ PBMCs from another donor and developed GVHD that was lethal (from day 17) in approximately 75% of the mice. Further, since we had observed high serum human IL-6 levels following MSC infusion in NSG mice in a prior study (44), we also assessed the impact of an i.p. injection of 4 mg tocilizumab, an anti-human IL-6 receptor antibody, 2 h before each MSC injection, in a sixth group of mice treated with 2 × 10^6^ UC-MSCs (UC2-T group). Unfortunately, as observed in the first cohort of mice, AT-MSCs induced injection-related mortality in 3 mice. Specifically, following the first AT-MSC injection on day 14, the 2nd mice receiving 2 × 10^6^ AT-MSCs died of probable pulmonary embolism (unfortunately no necropsy was performed to confirm pulmonary embolism); hence the 6 remaining mice received 1 × 10^6^ AT-MSCs. After the second injection, the 2 first mice given 2 × 10^6^ AT-MSCs died of probable pulmonary embolism; hence the 5 remaining mice received 1 × 10^6^ AT-MSCs for the second and the third injections, and there was no further acute mortality. Focusing on BM- and UC- MSCs, MSC therapy slightly delayed GVHD onset and increased median survival, but survival curves were not statistically different (median survival of 42, 59, and 59 days in the control, BM and UC2 groups, respectively). No dose effect was observed since survival was similar between mice treated with 1 or 2 × 10^6^ UC-MSCs (median survival 61 vs. 59 days, respectively). Finally, the adjunction of tocilizumab failed to enhance the efficacy of UC-MSC therapy (median survival 49 vs. 59 days in the UC2-T and UC2 groups, ns) (Figure 4B).

Given the high proportion of AT-MSC mice dying from MSC infusions, we elected to focus on BM-MSC and UC-MSC in the third cohort. Mice received 1 × 10^6^ PBMCs from a third donor which induced this time a relatively mild GVHD. They were treated with 1 × 10^6^ BM- or UC-MSCs, or the same volume of PBS alone (control group) on days 14, 18, and 22, as in the first cohort. Only 4 of the 24 mice died of GVHD (1, 1 and 2 in the control, BM and UC groups, respectively). One mouse in the control group died on day 6 of unexplained cause without sign of GVHD, and another mouse died right after the 2nd UC-MSC injection, probably of pulmonary embolism (Figure 4C). Blood samples were collected on day 19 (1 day after the second MSC infusion) and serum levels of human IL-6, IL-10, IFNγ, and TNFα were analyzed by Bio-Plex. We observed slightly higher human IL-6 serum levels in mice treated with UC-MSCs compared to controls, but not in mice treated with BM-MSCs (median 0.0, 0.4, and 3.2 pg/ml in the control, BM and UC groups, respectively). No differences in serum levels of human IFNγ (a marker of GVHD severity), TNFα and IL-10 were observed between the 3 groups at this early time-point (Supplementary Figure 3).

In order to further assess the impact of BM-MSCs or UC-MSCs on GVHD in the 3 cohorts combined, we built a Cox model adjusted for experiment (donor), mouse gender and mouse weight. We elected not to include AT-MSCs in the model given its high rate of injection-related mortality. We did not either include the data from the UC-Tocilizumab group. The multivariate model confirmed that intravenous MSC infusions failed to significantly increase survival (hazard ratio [HR] = 0.59, 95% CI 0.32–1.08; *P* = 0.087) (Supplementary Table 1). In a sensitivity analysis using the same adjustments as described above, we compared survival in control vs. each MSC group separately. The results for the BM-MSC vs. control comparison was HR = 0.63 (95% CI 0.30–1.34, *P* = 0.24) while the figures for the UC-MSC vs. control comparison was HR = 0.56 (95% CI 0.28–1.10, *P* = 0.09) (Supplementary Table 2). In concordance with these results, GVHD scores were not significantly lower in the MSC than in control mice (generalized estimating equation [GEE] estimate −0.7, 95% CI −1.8–0.3, *P* = 0.18) (Figures 4D–F).

### Characterization of Circulating Human Lymphoid Cells in Mice Treated With MSCs

We also analyzed circulating human lymphocytes in the peripheral blood of mice on days 28 and 42 post-transplantation. Proliferation of human lymphocytes was not significantly influenced by MSC therapy, as percentages of human CD45^+^ lymphoid cells, CD4/CD8 ratio and expression of Ki67 in CD4^+^ and CD8^+^ cells, were not significantly different in MSC groups compared to the control group (Supplementary Figure 4 including data from the 3 cohorts).

**Figure 5:**
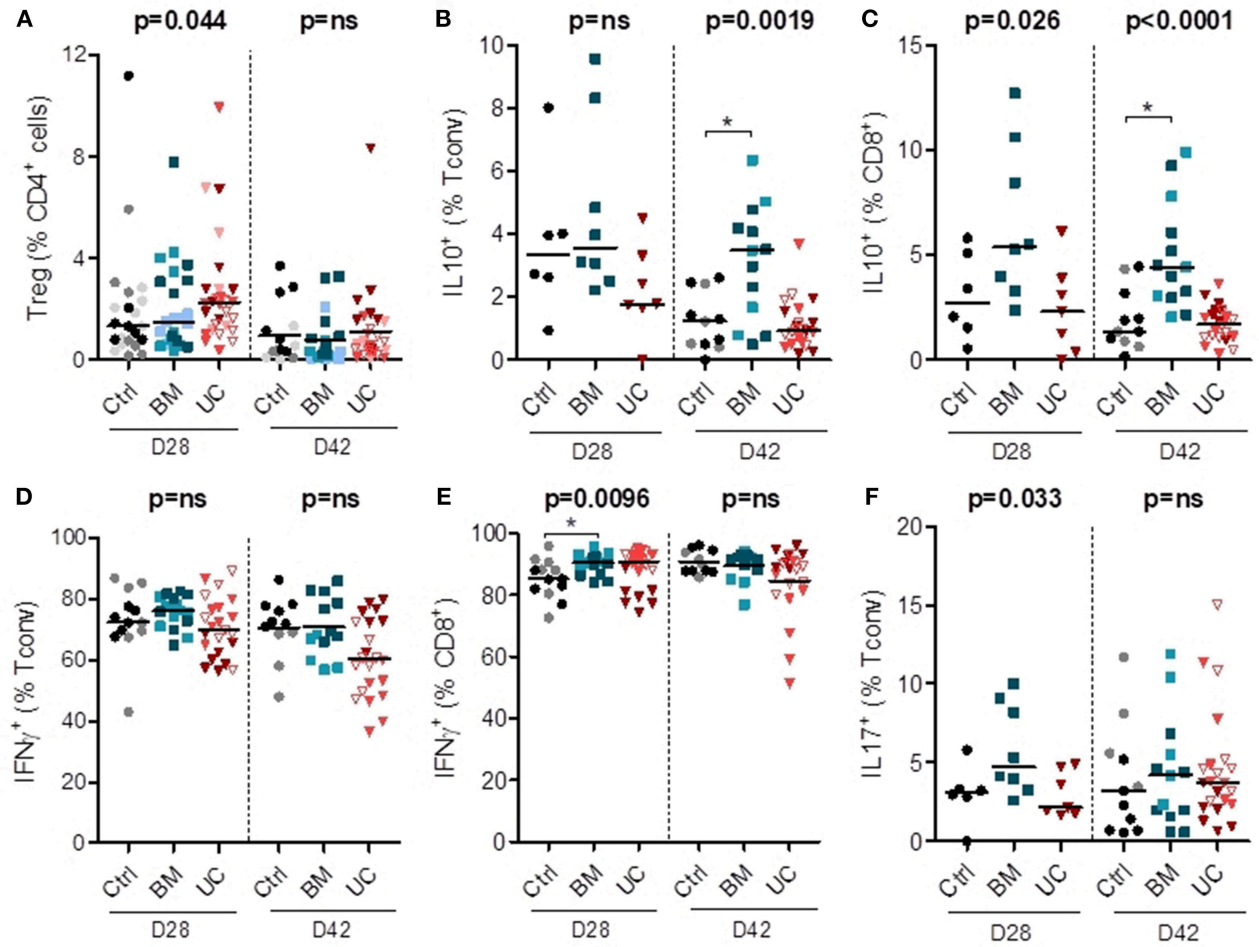
Circulating human lymphoid cell subsets in peripheral blood of mice on days 28 and 42 after transplantation. After 2 Gy total body irradiation, NSG-HLA-A2 mice were transplanted on day 0 with 1–1.5 × 10^6^ PBMCs and treated with 3 i.v. injections of 1–2 × 10^6^ MSCs derived from either BM or UC, or with PBS (control group) on days 14, 18, and 22. Peripheral blood samples were collected on days 28 and 42 after transplantation for flow cytometry analyses, including analyses of the proportions of **(A)** Tregs (CD25^+^FoxP3^+^) among CD4^+^ cells, **(B,C)** human Tconvs and CD8^+^ cells expressing IL-10, **(D–E)** human Tconv and CD8^+^ cells expressing IFNγ, and **(F)** human Tconv expressing IL-17. Data are presented as individual observations with median. Light, medium, and dark-colored symbols represent cohorts 1, 2, and 3, respectively, with empty symbols representing the lower dose UC group of the 2nd cohort. Global *p*-values (adjusted for experiment) are shown as well as comparisons between MSC groups and controls with Dunnett's *post-hoc* procedure (^*^p < 0.05). Prior logarithmic transformation was applied for Tregs on days 28 and 42, and for IL10^+^Tconv and IL10^+^CD8^+^ cells on day 42.

We also analyzed the impact of MSC therapy on Treg proportions (in all 3 cohorts) and intracellular cytokine expression in conventional (non-Treg) CD4^+^ (Tconv) and CD8^+^ cells (in cohorts 2 and 3). Treg frequencies remained low in all groups, although they were possibly slightly higher in the UC-MSC group on day 28 after transplantation (Figure 5A and Supplementary Figure 5). On day 28, there was also a trend toward a higher expression of IL-10 in CD8^+^ T cells (but not in CD4^+^ T cells) in the BM group (median 5.4% vs. 2.7 and 2.3% in the control and UC groups). On day 42, we observed a significant increase in the percentages of IL-10^+^ CD4^+^ and IL-10^+^ CD8^+^ cells in the BM group compared to the control group (IL-10^+^ CD4^+^: median 3.5 vs. 1.2%; IL-10^+^ CD8^+^: median 4.4 vs. 1.4%) (Figures 5B,C).

We also analyzed the impact of MSC therapy on pro-inflammatory IFNγ and IL-17 secreting cells (in cohorts 2 and 3). There was no significant impact of MSC therapy on the proportion of Th1 (IFNγ^+^ Tconv) cells on days 28 and 42 post-transplantation (Figure 5D and Supplementary Figure 5). We observed a higher proportion of IFNγ^+^ CD8^+^ cells in the BM group compared to the control group on day 28 post-transplantation (median 91 vs. 85%), while no significant difference was observed on day 42 (Figure 5E). We also observed a trend toward a higher proportion of Th17 (IL17^+^Tconv) cells in the BM group on day 28 (median 4.7 vs. 3.1 and 2.1% in control and UC groups, respectively), but no significant difference on day 42 post-transplantation (Figure 5F). Finally, there was no difference in the proportions of CD4^+^ and CD8^+^ cells expressing TNFα or IL-2 (Supplementary Figure 5).

Overall, these data suggest that UC-MSC therapy resulted in a trend toward a higher percentage of Tregs that nevertheless remained infrequent. BM-MSC therapy was associated with higher proportions and absolute numbers of IL-10^+^ cells, and also with a trend toward higher percentages of Th17 and IFNγ^+^CD8^+^ cells.

### *In vitro* Impact of MSCs on Coagulation

**Figure 6:**
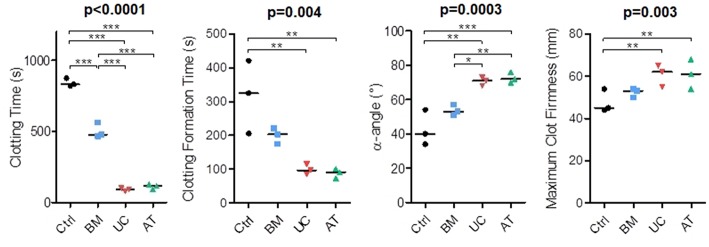
Rotational thromboelastometry (ROTEM) with blood incubated with MSCs. MSCs derived from BM, UC, or AT were incubated 10 min in citrated whole blood at a concentration of 10^6^ cells/ml, then CaCl_2_ (Star-TEM) was added to the sample and measurements of coagulation activation were made using ROTEM® (NATEM assay). Data are presented as individual observations with median. Global *p*-values (repeated measure ANOVA-1) are shown as well as two-by-two group comparisons with Bonferroni *post-hoc* procedure (^*^p < 0.05; ^**^p < 0.01; ^***^p < 0.001).

In our *in vivo* studies, several mice died right after IV injection of MSCs, mostly AT-MSCs (4 mice), but also UC-MSCs (1 mouse). Since the procoagulant activity of MSCs has been described (45) and since death by pulmonary embolism has been reported in MSC-injected mice (46, 47), we compared the procoagulant activity of MSCs of the 3 origins by performing rotational thromboelastometry (ROTEM). We used PBS as negative control. The experiment was repeated 3 times with MSCs from 2 donors and blood from 3 other healthy donors. We measured clotting time (CT; time from test start until a clot firmness amplitude of 2 mm is reached), clot formation time (CFT; time between 2 and 20 mm amplitude of the clotting signal), maximum clot firmness (MCF) and α-angle (angle between the baseline and a tangent to the clotting curve through the 2 mm amplitude point). We observed that BM-MSCs significantly reduced the clotting time when added to whole blood, but not as much as AT and UC-MSCs (median 831, 477, 117, and 92 s in the control, BM, AT, and UC groups, respectively). Similarly, we observed a shorter clotting formation time and a higher maximum clot firmness with AT and UC-MSCs compared to controls (median CFT 325, 203, 91, and 97 s and median MCF 45, 53, 61, and 62 mm in the control, BM, AT, and UC groups, respectively), with a significantly increased α-angle compared to control and BM-MSCs (median α-angle 40, 53, 72, and 71° in the control, BM, AT and UC groups, respectively) (Figure 6). These results show stronger coagulation activation by AT- and UC-MSCs compared to BM-MSCs. These data suggest that the higher mortality observed after AT-MSC infusion compared to UC-MSC infusion is the result of not only a higher induction of coagulation. Cell size and/or different expression of adhesion molecules might be involved.

## Discussion

Fifteen years after the first publication of a clinical success of MSC therapy in acute GVHD by Le Blanc et al. (48), the controversy about their efficacy still remains. The complexity of the mechanisms of action of MSCs, as well as their heterogeneity and plasticity depending on many factors such as their origin, culture conditions, or inflammatory environment, combined with the complex pathophysiology of GVHD and the heterogeneity of administration protocols and patient characteristics have contributed to the discrepancies between studies. Most clinical trials have used BM-MSCs, but fetal tissue-derived MSCs have the advantage of being readily available and easy to collect from a waste product. Moreover, even though they share many biological characteristics, MSCs from different origins differ in several instances, including phenotype, secreatome, or immunomodulatory properties. MSC from alternative origins might therefore be a better option than BM-MSCs in GVHD.

In this study, we compared the efficacy of BM-, AT- and UC-MSCs injected at day 14, 18, and 22 post-transplantation in a model of mixed xenogeneic and allogeneic GVHD in NSG-HLA-A2 mice (31). Indeed, although important differences remain between GVHD in humanized NSG mouse models and in humans (such as the GVHD-target organs, the lack of interaction between some mouse cytokines and human cells, or the absence of donor APC engraftment in the NSG mouse model), important key mechanisms of GVHD pathogenesis are shared in human and xenogeneic GVHD. These include expansion of T-cell clones that recognize genetic disparities with the recipient (including murine MHC and human HLA-A2 in our model) following activation of their TCR and co-stimulation with host APCs. This results in upregulation of IL-2/STAT5, mTOR and Aurora kinase A pathways, and differentiation toward effector T cells able to secrete high amounts of TNFα and IFNγ (31). Further, in contrast to mouse-to-mouse models of GVHD, humanized NSG(-HLA-A2) models take into consideration donor genetic diversity when different PBMC donors are used. We elected to infuse 1 × 10^6^ or 1.5 × 10^6^ PBMCs from non-HLA-A2 donors following 2 Gy irradiation, since we previously reported that infusion of 1 × 10^6^ PBMCs from non-HLA-A2 donors induced a moderate GVHD in that model while administration of 2 × 10^6^ PBMCs resulted in very severe GVHD (31). Indeed, infusion of the same dose of PBMCs from non-HLA-A2 donors consistently results in dramatically worse GVHD in NSG-HLA-A2 than in NSG mice (in which 2 × 10^6^ to 5 × 10^6^ PBMCs are usually infused to induce GVHD when NSG mice are previously irradiated (31, 49–52). While the PBMC dose infused appeared adequate for experiment (donor) #1 and #2, it was suboptimal with the donor for the 3rd experiment since most of the control mice it that group survived beyond day 90.

The main observation of our study was that MSC infusions failed to significantly prevent GVHD-related mortality. We cannot exclude that this was due to the sample size since the HR for mortality was in favor of (UC- and BM-) MSC therapy. However, the number of mice studied (a total of 24 control and 56 UC- or BM-MSC mice) was quite decent and several anti-GVHD prevention strategies proved to be efficient in NSG (36, 49, 53) or NSG-HLA-A2 mice (53) using fewer mice per arm. Interestingly, despite their different *in vitro* ability to inhibit T cells, we did not observe significant differences in term of GVHD prevention by BM-, UC- or AT-MSCs. However, one may argue that UC-MSCs appear more efficient than BM-MSCs while the efficacy of AT-MSCs was difficult to establish since several mice died of probable pulmonary embolism immediately after injection.

The dose and timing of infusion can always be discussed. However, we do not believe that the MSC dose was insufficient, since we infused a much higher dose of MSCs per kilogram compared to human studies, and since no differences were observed between the two UC-MSC dose groups in the second cohort. Although one could argue that the dose of MSC infused might have been too high, prior experimental studies have demonstrated better GVHD control with higher doses of MSC administered (54, 55). We elected to infuse MSCs from day 14, when mice showed the first signs of GVHD. Indeed, several previous studies showed the inefficacy of resting unmanipulated MSCs when infused before GVHD onset, while IFNγ primed MSCs prevented GVHD (39, 56). We hypothesized that the high circulating levels of TNFα and IFNγ on day 14 in the NSG-HLA-A2 model (31) could activate MSCs *in vivo* and increase their efficacy (as observed *in vitro*). Although starting MSC injection at day 14 might have been too late to prevent the aggressive and already engaged GVHD process, most trials of MSC therapy for acute GVHD have included patients in active (mostly steroid-refractory) acute GVHD.

Several prior articles have assessed the ability of BM- or cord blood (CB)-MSC to prevent or treat xenogeneic GVHD, although none compared the different MSC sources (44, 55–62) (Table 1). These studies differ in terms of source of MSC, schedule of MSC infusion as well as type/number/route of injection of human PBMCs. While some observed longer survival with MSCs (61), several others failed to demonstrate a significant benefit of MSCs as treatment of xenogeneic GVHD, as observed in the current study (44, 57).

**Table 1:** Main prior studies of MSC as prevention/treatment of xenogeneic GVHD.

**References**	**Xenogeneic mouse model**	**MSC source, dose, and schedule of administration**	**Main observations**
**PREVENTION**			
Tisato et al. (57)	NOD/SCID, TBI 2.5 Gy, 20 × 10^6^ hPBMCs IV	3 × 10^6^ CB-MSCs IV, day 0	No change in weight loss and human T-cell expansion.
		3 × 10^6^ CB-MSCs IV, days 0, 7, 14, and 21	Decreased T-cell expansion, no GVHD development.
Gregoire-Gauthier et al. (58)	NSG, TBI 3 Gy, 10 × 10^6^ hPBMCs IP	1 × 10^6^ CB-MSCs IV, day 0	Significant increase in survival and reduction of clinical signs of GVHD.
Bruck et al. (44)	NOD/SCID, TBI 3 Gy + aASGM1 Ab IP, 200 × 10^6^ hPBMCs IP	2 × 10^6^ BM-MSCs IV or IP, day 0	No significant increase in survival.
	NSG, TBI 2.5 Gy, 30 × 10^6^ hPBMCs IP	3 × 10^6^ BM-MSCs IP, days 0, 7, 14, and 21	Slight survival advantage.
		3 × 10^6^ IFNγ-BM-MSCs IP, days 0, 7, 14, and 21	No significant increase in survival.
		3 × 10^6^ BM-MSCs IV, days 0, 7, and 14	No significant increase in survival.
Tobin et al. (56)	NSG, TBI 2.4 Gy, 6.3 × 10^5^ hPBMCs/g BW	4.4 × 10^4^ BM-MSCs/g BW, IV, day 7	Increased survival, reduction of liver and gut pathology.
		4.4x10^4^ IFNγ-BM-MSCs/g BW, IV, day 0	Increased survival, reduced liver and gut pathology, and serum level of TNFα.
Jang et al. (59)	NSG, TBI 2 Gy, 1 × 10^6^ hPBMCs IV	5 × 10^5^ CB-MSCs IV, day 0 or days 0, 7, and 14	No significant increase in survival.
		5 × 10^5^ CB-MSCs IV, days 0, 3 and 6	Increased survival, reduced tissue damage, lymphocyte infiltration, and GVHD clinical scores.
Girdlestone et al. (55)	BALB/c RAG2^−/−^ (γc)^−/−^, TBI 4 Gy, 15 × 10^6^ hPBMCs IV	0.5 × 10^6^ UC-MSCs IV, day 8	No significant increase in survival.
		2 × 10^6^ UC-MSCs IV, day 8	Trend toward a longer survival.
		0.5 × 10^6^ rapamycin-UC-MSCs IV, day 8	Increased survival, lower proportion of human cells in the spleen.
Kim et al. (60)	NOD/SCID, TBI 3.2 Gy, 20 × 10^6^ hPBMCs IV	1 × 10^6^ BM-MSCs (normoxia or 1% O_2_) IV, days 0 and 7 or days 0, 3, and 6	Increased survival, reduced GVHD symptoms (no difference between normoxia and hypoxia).
**TREATMENT**			
Tisato et al. (57)	NOD/SCID, TBI 2.5 Gy, 20 × 10^6^ hPBMCs IV	3 × 10^6^ CB-MSCs IV 4 times every 3 days at GVHD onset	No change in weight loss and human T-cell expansion.
Jang et al. (59)	NSG, TBI 2 Gy, 1 × 10^6^ hPBMCs IV	5 × 10^5^ CB-MSCs IV, either day 18, days 18, 21 and 24, or days 18, 25, and 32	Increased survival, reduced weight loss, clinical scores, tissue damage, and lymphocyte infiltration.
Amarnath et al. (61)	NSG, 5 × 10^6^ Th1 cells + 3 × 10^6^ monocytes IV	2 × 10^6^ BM-MSCs IV, days 22, 26, and 30	Increased survival, reversal of cutaneous GVHD and weight loss, decreased proportion of human Th1 cells in the spleen.
Ma et al. (62)	NOD/SCID, CY + aASGM1 Ab IP, 10 × 10^6^ hPBMCs IV	1 × 10^6^ placenta-derived MSCs IV, day 11	Increased survival, reduced weight loss, reduced lung and intestinal damage, increased serum level of TGFβ, decreased serum level of IL-6 and IL-17, reduced Th17/Tr1 ratio in spleen and liver.

*NOD/SCID, Non-obese diabetic/severe combined immunodeficiency; TBI, total body irradiation; hPBMCs, human peripheral blood mononuclear cells; IV, intravenous; CB, cord blood, MSCs, mesenchymal stromal cells; GVHD, graft- vs.-host disease; aASGM1 Ab, anti-asialo GM1 antibody; IP, intraperitoneally; BM, bone marrow; NSG, NOD-scid IL-2Rγnull; IFN, interferon; g BW, gram of body weight UC, umbilical cord; Th1, T helper 1; CY, Cyclophosphamide; Tr1, type 1 regulatory T cells (CD4^+^IL10^+^)*.

Another observation of our *in vivo* studies was that intravenous infusion of 1 × 10^6^ UC-MSCs was followed by a peak of serum IL-6 while infusion of the same number of BM-MSCs did not. Our team previously showed that an i.p. infusion of 3 × 10^6^ BM-MSCs resulted in a peak of serum IL-6 (44), but it is possible that the rise in IL-6 following the infusion of a smaller amount of MSCs did not reach the detection limit of the technique. This finding is consistent with several *in vitro* studies that have demonstrated a higher secretion of IL-6 from UC-MSCs compared to BM-MSCs (63). Unfortunately, co-treatment with the anti-IL6R tocilizumab did not improve survival in mice treated with UC-MSCs, suggesting that this pro-inflammatory signal following UC-MSC infusions does not lessen their efficacy.

Flow cytometry analyses performed at day 28 post-transplantation revealed a trend toward an increased proportion of Tregs in mice treated with UC-MSCs, while BM-MSC therapy was associated with an increased proportion of IL10^+^ lymphocytes, but also a trend toward an increased proportion of Th17 cells [whose role in xenogeneic GVHD in humanized mouse models is increasingly demonstrated (37, 64)] and IFNγ^+^CD8^+^ cells. Most of these differences were lost at day 42, confirming the limited long-term effects of MSC therapy in this GVHD model. However, a survival bias cannot be ruled out. Further, it should be emphasized that Treg frequencies, even in UC-MSC mice, remained low (in the range of 2.5%) compared to what has been achieved in this model with Treg-promoting therapies such as azacitidine (53).

In *in vitro* co-culture, we observed that resting BM- and AT-MSCs inhibited PBMC proliferation induced by anti-CD3/CD28 beads more potently than resting UC-MSCs. However, UC-MSC efficacy was significantly enhanced by priming with IFNγ and TNFα. Lymphocyte proliferation was inhibited by primed BM- and AT-MSCs to a little higher extent than by primed UC-MSCs, but the difference was not statistically significant. Conflicting results have been reported in the literature about the relative potency of BM-, AT- and UC-MSCs to inhibit T-cell proliferation, which seems to depend on the proliferative stimulus and priming of MSCs (65–67). In our murine model, this ability to inhibit lymphocyte proliferation did not translate into a reduction of CD45^+^ cell chimerism or Ki67 expression in CD4^+^ and CD8^+^ cells in MSC groups compared to controls, possibly because MSCs were infused after the early T-lymphocyte expansion phase. Similarly, a study in patients showed that Ki67 expression by lymphocytes was not modulated by MSC infusion (68).

MSCs also modulate the lymphocyte activation status. In a retrospective study on BM-MSC therapy for GVHD, MSC-treated patients had lower proportions of HLA-DR^+^CD4^+^ cells at day 90 and of HLA-DR^+^CD8^+^ cells at day 180 post-MSC infusions (68). We studied lymphocyte activation by anti-CD3/CD28 beads in co-culture with MSCs, and observed that UC- and AT-MSCs, whether resting or primed with IFNγ and TNFα, induced the most potent down-regulation of HLA-DR on CD4^+^ and CD8^+^ cells. Importantly, co-culture of PBMCs with primed BM-MSCs resulted in a higher expression of the early activation marker CD69 and in a rapid upregulation of HLA-DR 24 h after activation of PBMCs. Similarly, other authors reported an early and transient upregulation of the co-stimulatory receptor CD28 on PHA-stimulated lymphocytes in co-culture with IFNγ-primed BM-MSCs but not with resting BM-MSCs or either resting or primed UC-MSCs (67). Therefore, unlike UC- and AT-MSCs, BM-MSCs in an inflammatory environment seem to induce a rapid pro-inflammatory reaction in contact with PBMCs before exerting their immunosuppressive properties. Accordingly, we observed at day 28 a higher expression of HLA-DR on Tconv cells in the BM group compared to the UC group in the third cohort.

The effects of MSCs on T lymphocytes are thought to combine not only suppression of pro-inflammatory cells but also induction of Tregs. In a retrospective study, BM-MSC treated patients had a higher proportion of Tregs at days 30 and 90 and of IL10^+^CD4^+^ cells at day 90, a lower percentage of Th17 cells at day 30, and a lower serum IFNγ/IL-4 ratio (68). As mentioned above, in our murine model there was a trend toward a higher proportion of Tregs with UC-MSCs, while BM-MSCs induced higher levels of IL10^+^CD4^+^ cells, but also tended to induce higher levels of Th17 cells. This is consistent with our observation that, *in vitro*, UC-MSCs most efficiently induced Tregs and inhibit Th17 cells, while BM-MSCs induced higher proportions of CD4^+^IL10^+^ cells. AT-MSCs induced both Tregs and CD4^+^IL10^+^ cells. We also observed a lower proportion of Th1 cells on day 28 in mice treated with UC-MSC in the 3rd cohort. Direct comparisons of the effects of MSCs of different origins on T-lymphocyte subsets are scarce (29). UC-MSCs were shown to induce Tregs more potently than BM-MSCs *in vitro* (69) or to the same extent in a rat model of sepsis (70). MSCs could also induce other types of regulatory T cells although conflicting data have been reported concerning the mechanisms involved (71) and the comparison between BM- and UC-MSCs (67, 72). Regarding Th17 cells, most studies demonstrated a suppressive effect of MSCs, but no comparison between BM-, UC- and AT-MSC potency has been reported. We did not observe a reduction in the proportion of CD4^+^ cells secreting IFNγ in co-culture with MSCs in this experimental setting. This is consistent with prior observations by De Witte et al. who observed a decrease in the percentage of CD4^+^ T cells containing intracellular IFNγ only at higher MSC/PBMC ratios (73), while Ribeiro et *al*. observed an upregulation of T-bet mRNA with BM-, UC- and AT-MSCs (74).

Finally, we also demonstrated that BM-MSCs are far less procoagulant than UC- and AT-MSCs. However, despite a similar activation of coagulation *in vitro*, UC-MSCs resulted in only 1 death after infusion in mice, while 4 mice infused with AT-MSCs died, suggesting that other factors than their potential to activate coagulation may be involved. Cell size and expression of adhesion receptors are key factors in pulmonary cell trapping, so the higher size of AT-MSCs might be at least partially responsible. Similar results have been observed with murine AT-MSCs (46) and human decidual stromal cells (DSCs) (47). In humans, while BM-MSCs have demonstrated their safety, there are a few cases of thrombotic events following infusions of AT-MSCs (75). MSCs activate coagulation through tissue factor expression, which is expressed at higher levels on AT- and placenta-derived MSCs (45). Heparin infusion was shown to prevent this effect in a porcine model of acute myocardial infarction (76). BM-MSCs have also been used to treat hemorrhages in a few patients (gastro-intestinal bleeding, hemorrhagic cystitis) (77). The higher procoagulant effects of UC- and AT-MSCs might be of interest in these settings.

There are limitations in our study. First, we did not include placenta-derived decidua stromal cells (DSCs) among the sources of stromal cells we compared. Indeed, recent studies in humans have suggested that these cells could be more potent than BM-MSCs to treat acute GVHD (78). Secondly, it has been recently demonstrated that monocytes are important for induction of Tregs by MSCs *in vitro* (79). Moreover, in a murine model of GVHD, apoptosis of MSCs induced by cytolytic cells (NK and CD8^+^ cells) and phagocytosis of apoptotic MSCs by macrophages were necessary to MSC-induced immunosuppression (80). Given that human monocytes / macrophages do not engraft in NSG mice, it is possible that the humanized NSG mouse model is not the most suitable model to study the impact of MSCs on GVHD. However, one could argue that NSG mice have nevertheless functional autologous macrophage and dendritic cells that are able to modulate the activity of infused human PBMCs (52). Other potential limitations of this study are the fact that the timing of the first MSC administration might have been too late, when irreversible immunological mechanisms were already in place, or that the GVHD induced by injection of HLA-A2-negative PBMC in NSG-HLA-A2 mice (combining xeno- and allo- reactions) is perhaps too strong to be counterbalanced by immune regulatory mechanisms. However, one could also argue that the dose of PBMC infused in our study (1–1.5 × 10^6^ PBMC/mice) was rather in the lower range of what has been used to induce GVHD in NSG or NSG-HLA-A2 mice. Finally, in order to take into consideration genetic variability, we elected to include different PBMC and MSC donors for each experiment to increase the robustness of our results. In order to tackle the variability issue, we performed multivariate Cox models that confirmed a significant impact of the PBMC donors (as previously reported (36, 37), no impact of mouse gender and weight, as well as no impact of MSC infusion on survival (Supplementary Table 1).

In summary, our data show that BM-, AT-, and UC-MSCs have differential effects on immune cells. UC-MSCs seem to promote a more “resting” phenotype in lymphocytes, with a potent down-regulation of HLA-DR, a higher induction of Tregs, and a decreased proportion of pro-inflammatory cells. On the other hand, BM-MSCs promote higher IL10 expression by T lymphocytes, but also more inflammatory features, especially when primed in inflammatory conditions. *In vivo*, both BM- and UC-MSCs failed to significantly delay GVHD mortality. Other types of MSCs derived from fetal membranes seem promising for GVHD therapy, and it would be interesting to compare them to BM- and UC-MSCs in preclinical studies. Also, gene modification of MSCs (for example in order to force secretion of regulatory cytokines such as IL-10) might increase their ability to protect against GVHD (81). Finally, the procoagulant effects of UC-MSCs and AT-MSCs should be taken into consideration in further clinical studies.

## Data Availability

All datasets generated for this study are included in the manuscript and/or the Supplementary Files.

## Author Contributions

CG, CR, MH, LD, and SD performed the experiments. CG and FB analyzed and interpreted the data. LS made the statistical analyses. SV isolated and cultured the AT-MSCs and CL and AB the BM-MSCs. LB, GE, SS, and YB helped in data interpretation. CG and FB designed the research and wrote the article. All authors edited the manuscript and approved its final version.

### Conflict of Interest Statement

FB has received travel grants from Celgene, Abbvie, Novartis, and Sanofi. The remaining authors declare that the research was conducted in the absence of any commercial or financial relationships that could be construed as a potential conflict of interest.
