# Supplementary material for: Comparison of Mesenchymal Stromal Cells From Different Origins for the Treatment of Graft-vs.-Host-Disease in a Humanized Mouse Model
Source: Front Immunol. 2019 Apr 2;10:619. doi: 10.3389/fimmu.2019.00619 (PMC6454068; doi:10.3389/fimmu.2019.00619)
Supplement: Supplementary file 1 [file Data_Sheet_1.docx]

**Supplementary table 1**

| Parameter |  | DF | Parameter Estimate | Standard Error | Chi-Square | Pr > ChiSq | Hazard Ratio | 95% Hazard Ratio Confidence Limits | |
| --- | --- | --- | --- | --- | --- | --- | --- | --- | --- |
| Group | MSC | 1 | -0.53145 | 0.31092 | 2.9217 | 0.0874 | 0.588 | 0.320 | 1.081 |
| Donor/Experiment | 2 | 1 | -0.19200 | 0.41399 | 0.2151 | 0.6428 | 0.825 | 0.367 | 1.858 |
| Donor/Experiment | 3 | 1 | -1.73837 | 0.59331 | 8.5847 | 0.0034 | 0.176 | 0.055 | 0.562 |
| Mouse gender | F | 1 | 0.57356 | 0.49326 | 1.3521 | 0.2449 | 1.775 | 0.675 | 4.666 |
| Mouse weight |  | 1 | -0.02401 | 0.09318 | 0.0664 | 0.7966 | 0.976 | 0.813 | 1.172 |

**Impact of MSCs (vs control) on GVHD in the 3 cohorts combined.**

Cox model adjusted for experiment (donor), mouse gender and mouse weight.

DF = Degrees of freedom; F = female.

**Supplementary table 2**

| Parameter |  | DF | Parameter Estimate | Standard Error | Chi-Square | Pr > ChiSq | Hazard Ratio | 95% Hazard Ratio Confidence Limits | |
| --- | --- | --- | --- | --- | --- | --- | --- | --- | --- |
| Group | BM | 1 | -0.45244 | 0.38211 | 1.4020 | 0.2364 | 0.636 | 0.301 | 1.345 |
| Group | UC | 1 | -0.58496 | 0.34866 | 2.8148 | 0.0934 | 0.557 | 0.281 | 1.103 |
| Donor/Experiment | 2 | 1 | -0.15135 | 0.43086 | 0.1234 | 0.7254 | 0.860 | 0.369 | 2.000 |
| Donor/Experiment | 3 | 1 | -1.71162 | 0.59831 | 8.1840 | 0.0042 | 0.181 | 0.056 | 0.583 |
| Mouse gender | F | 1 | 0.62862 | 0.51974 | 1.4629 | 0.2265 | 1.875 | 0.677 | 5.193 |
| Mouse weight |  | 1 | -0.03401 | 0.09828 | 0.1197 | 0.7293 | 0.967 | 0.797 | 1.172 |

**Impact of BM- or UC-MSCs (vs control) on GVHD in the 3 cohorts combined.**

Cox model adjusted for experiment (donor), mouse gender and mouse weight.

DF = Degrees of freedom; F = female.

**Supplementary Figure 1**

**
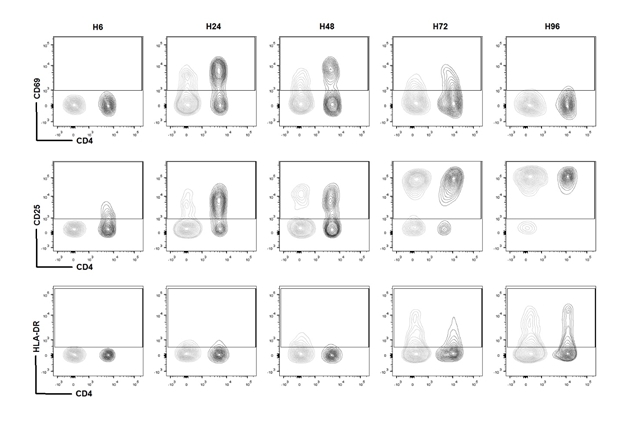

Representative plots of CD69, CD25 and HLA-DR upregulation on CD4^+^ and CD8^+^ cells in control condition *in vitro*.**

PBMCs were cultured with or without MSCs in the presence of anti-CD3/CD28 microbeads for 4 days, at a MSC/PBMC ratio of 1/10. Expression of CD69, CD25 and HLA-DR on CD4^+^ and CD8^+^ lymphocytes was analyzed by FACS after 6, 24, 48, 72 and 96 hours.

**Supplementary Figure 2**


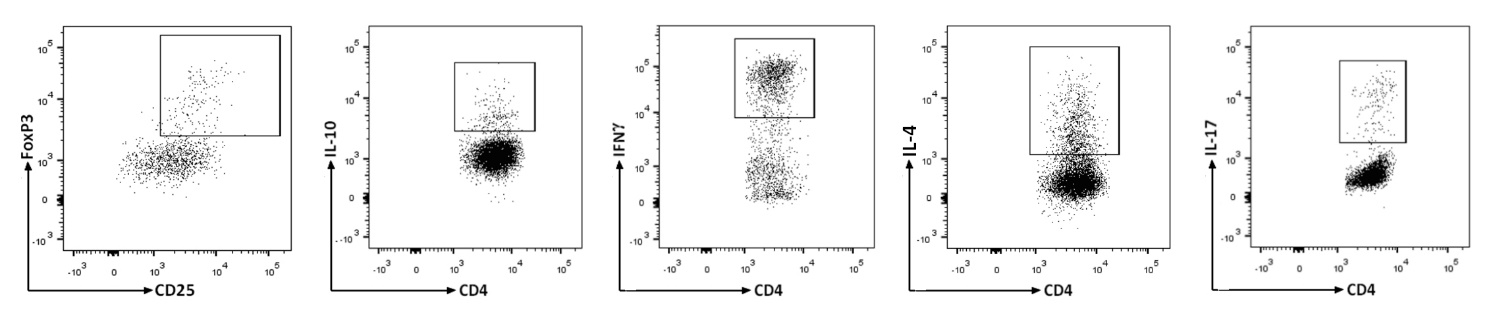

**Representative plots of Tregs (CD4^+^CD25^+^FoxP3^+^) and expression of IL-10, IFNγ, IL-4 and IL-17 on CD4^+^ cells in control condition *in vitro*.**

PBMCs were cultured with or without MSCs in the presence of anti-CD3/CD28 microbeads (and IL-2 for Treg analyses) for 7 days, at a MSC/PBMC ratio of 1/10. Proportions of Tregs (CD4^+^CD25^+^FoxP3^+^), Th1 (IFNγ^+^), Th2 (IL-4^+^), Th17 (IL-17^+^) and IL10^+^ cells were evaluated at day 7 by FACS.

**Supplementary Figure 3**

**
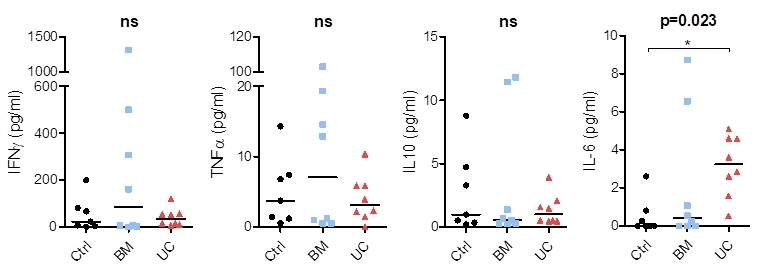
**

**Cytokine levels in peripheral blood of mice one day after MSC injection (data from cohort 3).**

After 2 Gy total body irradiation, NSG-HLA-A2 mice were transplanted on day 0 with 1x10^6^ PBMCs and treated with 3 i.v. injections of 1x10^6^ MSCs derived from either BM or UC, or with PBS (control group) on days 14, 18 & 22 (cohort 3). Peripheral blood samples were collected on day 19 after transplantation (one day after the second MSC infusion) and serum was stored at -80°C, and then thawed for Bio-Plex analysis. Data are presented as individual observations with median. Global p-values (repeated measure ANOVA-1) are shown as well as comparisons between MSC groups and controls with Dunnett’s post-hoc procedure (*p <0 .05). Prior square root transformation was applied for INFγ and IL-6, and prior logarithmic transformation was applied for TNFα and IL-10.

**Supplementary Figure 4**

**
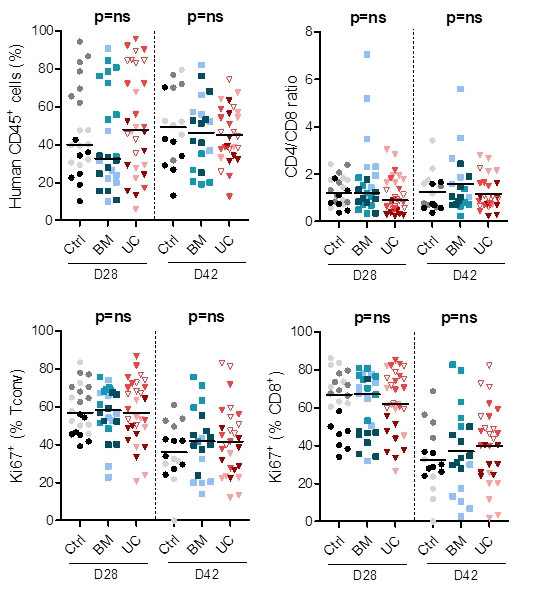
**

**Circulating human lymphoid cells in peripheral blood of mice on days 28 and 42 after transplantation.**

After 2 Gy total body irradiation, NSG-HLA-A2 mice were transplanted on day 0 with 1-1.5x10^6^ PBMCs and treated with 3 iv injections of 1-2x10^6^ MSCs derived from either BM or UC, or with PBS (control group) on days 14, 18 & 22. Peripheral blood samples were collected on days 28 and 42 after transplantation for flow cytometry analyses, including analyses of the proportion of human CD45 cells, CD4^+^/CD8^+^ human T-cell ratio, and proportions of human Tconv and CD8^+^ T cells expressing Ki67. Data are presented as individual observations with median. Light, medium and dark-colored symbols represent cohorts 1, 2 and 3, respectively, with empty symbols representing the lower dose UC group of the 2^nd^ cohort. Global p-values (adjusted for experiment) are shown. Prior logarithmic transformation was applied for human CD45^+^ cells at day 28 and CD4/CD8 ratio on days 28 and 42.

**Supplementary Figure 5**

**
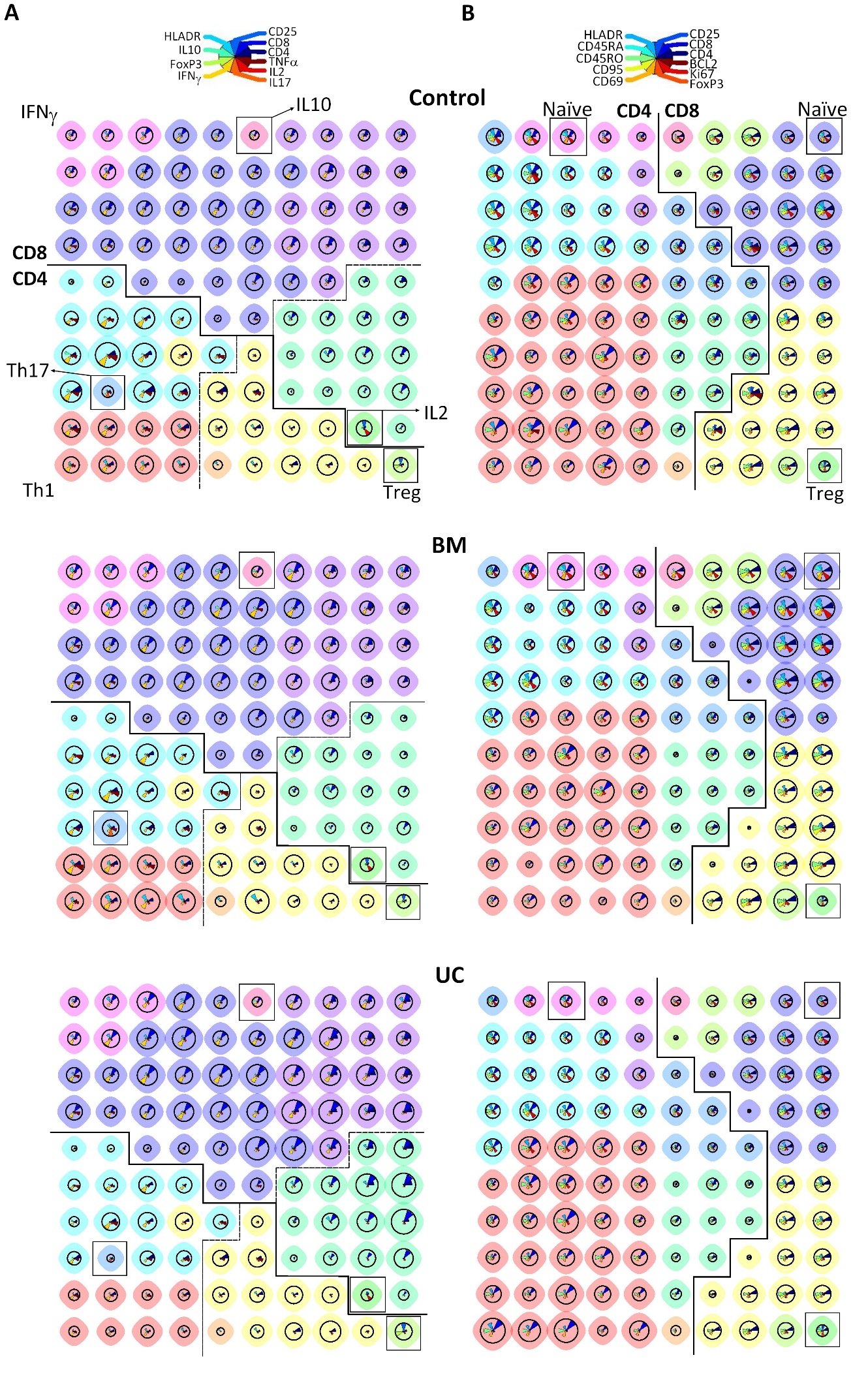
**

**Computational analysis of circulating human lymphoid cells in peripheral blood of mice on days 28 and 42 after transplantation (data from cohort 3).**

After 2 Gy total body irradiation, NSG-HLA-A2 mice were transplanted on day 0 with 1x10^6^ PBMCs and treated with 3 i.v. injections of 1x10^6^ MSCs derived from either BM or UC, or with PBS (control group) on days 14, 18 & 22 (cohort 3). Peripheral blood samples were collected on days 28 (n=21) (A) and 42 (n=22) (B) after transplantation for flow cytometry analyses. Data were compensated, then human CD45^+^ cells were manually gated with FlowJo v10, concatenated within the same group and analyzed with FlowSOM (1). Cells were clustered into 100 distinct clusters based on their expression of the different markers. Clusters are represented by star charts, indicating the relative expression for each of the different markers, and organized in a grid; the size of the nodes represents the proportion of cells in the cluster. An automatic meta‐clustering of the FlowSOM nodes in 12 populations was performed and is indicated by the background color of the nodes.

Reference:

1. Van Gassen S, Callebaut B, Van Helden MJ, Lambrecht BN, Demeester P, Dhaene T, et al. FlowSOM: Using self-organizing maps for visualization and interpretation of cytometry data. *Cytometry A* (2015) 87(7):636-45. doi: 10.1002/cyto.a.22625. PubMed PMID: 25573116.
